# Measles Virus Hemagglutinin Protein Establishes a Specific Interaction With the Extreme N-Terminal Region of Human Signaling Lymphocytic Activation Molecule to Enhance Infection

**DOI:** 10.3389/fmicb.2020.01830

**Published:** 2020-08-14

**Authors:** Fumio Seki, Yuta Yamamoto, Hideo Fukuhara, Kazue Ohishi, Tadashi Maruyama, Katsumi Maenaka, Hiroaki Tokiwa, Makoto Takeda

**Affiliations:** ^1^Department of Virology 3, National Institute of Infectious Diseases, Tokyo, Japan; ^2^Department of Chemistry, Rikkyo University, Tokyo, Japan; ^3^Faculty of Pharmaceutical Sciences, Hokkaido University, Sapporo, Japan; ^4^Faculty of Engineering, Tokyo Polytechnic University, Atsugi, Japan; ^5^School of Marine Biosciences, Kitasato University, Tokyo, Japan

**Keywords:** measles virus, signaling lymphocytic activation molecule, receptor, hemagglutinin, extreme N-terminal

## Abstract

Measles virus (MV) is a human pathogen that is classified in the genus *Morbillivirus* in the family *Paramyxoviridae* together with several non-human animal morbilliviruses. They cause severe systemic infections by using signaling lymphocytic activation molecule (SLAM) and poliovirus receptor-like 4 expressed on immune and epithelial cells, respectively, as receptors. The viral hemagglutinin (H) protein is responsible for the receptor-binding. Previously determined structures of MV-H and SLAM complexes revealed a major binding interface between the SLAM V domain and MV-H with four binding components (sites 1–4) in the interface. We studied the MV-H and human SLAM (hSLAM) complex structure in further detail by *in silico* analyses and determined missing regions or residues in the previously determined complex structures. These analyses showed that, in addition to sites 1–4, MV-H establishes a unique interaction with the extreme N-terminal region (ExNTR) of hSLAM. The first principles calculation-based fragment molecular orbital computation method revealed that methionine at position 29 (hSLAM-Met29) is the key residue for the interaction. hSLAM-Met29 was predicted to establish a CH-π interaction with phenylalanine at position 549 of MV-H (MVH-Phe549). A cell-cell fusion assay showed that the hSLAM-Met29 and MVH-Phe549 interaction is important for hSLAM-dependent MV membrane fusion. Furthermore, Jurkat cell lines expressing hSLAM with or without Met29 and recombinant MV possessing the H protein with or without Phe549 showed that the hSLAM-Met29 and MVH-Phe549 interaction enhanced hSLAM-dependent MV infection by ~10-fold. We speculate that in the evolutionary history of morbilliviruses, this interaction may have contributed to MV adaptation to humans because this interaction is unique for MV and only MV uses hSLAM efficiently among morbilliviruses.

## Introduction

Despite the availability of highly effective vaccines, measles remains a major cause of human mortality and morbidity. The causative agent is measles virus (MV), which belongs to the genus *Morbillivirus* in the family *Paramyxoviridae*. MV causes a systemic infection by infecting several types of lymphoid and myeloid cells in the immune system and epithelia of multiple organs/tissues. To achieve this, MV uses two types of cell surface molecules as receptors, signaling lymphocytic activation molecule (SLAM) and poliovirus receptor-like 4 (PVRL4 or nectin-4) expressed on immune and epithelial cells, respectively (Tatsuo et al., 2000b; Muhlebach et al., 2011; Noyce et al., 2011). MV has two types of glycoprotein spikes on the envelope, the hemagglutinin (H) and fusion (F) proteins. The H protein of MV (MV-H) binds to the receptor on a target cell and the F protein mediates membrane fusion between the virus envelope and the plasma membrane of the target cell to initiate infection. Five non-human animal morbilliviruses, rinderpest virus (RPV), peste des petits ruminants virus (PPRV), cetacean morbillivirus (CeMV), phocine distemper virus (PDV), and canine distemper virus (CDV), also use the host SLAM and PVRL4 as receptors (Takeda et al., 2020). This receptor-usage is a common characteristic of morbilliviruses. The amino acid sequence of PVRL4 is highly conserved across species, while that of SLAM varies significantly (Takeda et al., 2020). Previous studies have suggested that this SLAM variation may create a cross-species barrier among morbilliviruses (Tatsuo et al., 2001; Ohishi et al., 2019). However, accumulated evidence shows that in many cases non-host animal SLAMs function efficiently as receptors for different morbilliviruses (Seki et al., 2020; Takeda et al., 2020). One exception is human SLAM (hSLAM). When compared with cetacean (dolphin), phocine (seal), and canine (dog) SLAMs, which function as a receptor for all tested morbilliviruses (MV, CeMV, PDV, and CDV), hSLAM is uniquely specific for MV (Takeda et al., 2020). hSLAM cannot be used by CeMV, PDV, or CDV (Bieringer et al., 2013; Seki et al., 2020; Takeda et al., 2020). RPV may use hSLAM but less efficiently than MV (Tatsuo et al., 2001; Takeda et al., 2020). Thus, MV may have a unique interaction mechanism with hSLAM. The V domain of hSLAM is critically important for MV binding (Ono et al., 2001b). A β-sheet structure, composed of four β strands, in the V domain (V domain β-sheet) provides the major binding interface between SLAM and MV-H (Hashiguchi et al., 2011). Crystal structure data (Hashiguchi et al., 2011) show four binding components (sites 1–4) between SLAM and MV-H (Hashiguchi et al., 2011). In this study, an additional important interaction between hSLAM and MV H protein (MV-H) was identified. The interaction was established using the extreme N-terminal region (ExNTR) of hSLAM. Our functional analysis of expressed proteins and infectious viruses shows that this interaction significantly enhances the ability of MV to use hSLAM. Because this interaction was unique for MV, it may have contributed to MV adaptation to humans.

## Materials and Methods

### Cells

BHK/T7-9 cells constitutively expressing T7 RNA polymerase (Ito et al., 2003) were maintained in Dulbecco’s modified Eagle’s medium (DMEM) supplemented with 10% fetal bovine serum (FBS). Vero.DogSLAMtag and Vero/hSLAM were reported previously (Ono et al., 2001a; Seki et al., 2003) and were maintained in DMEM supplemented with 7% FBS and 0.5 mg/ml geneticin (G418; Gibco). The expression plasmids for the dual split protein (DSP) system (pRL-DSP_1–7_ and pRL-DSP_8–11_) were kindly provided by Dr. Z. Matsuda (The University of Tokyo; Kondo et al., 2010; Ishikawa et al., 2012). The region including the SV 40 enhancer/promoter and coding region of neomycin phosphotransferase was obtained from pCI-neo (Promega) and inserted into pRL-DSP_1–7_ and pRL-DSP_8–11_, generating pRL-DSP_1–7_-neo and pRL-DSP_8–11_-neo, respectively. Chinese hamster ovarian (CHO)/DSP_1–7_ and CHO/DSP_8–11_ cells were generated by transfecting CHO cells with pRL-DSP_1–7_-neo and pRL-DSP_8–11_-neo, respectively, and were selected in Roswell Park Memorial Institute (RPMI) medium supplemented with 7% FBS and 0.5 mg/ml geneticin. Jurkat cells were maintained in RPMI medium supplemented with 10% FBS. Jurkat/hSLAM, Jurkat/hSLAM-M29S, and Jurkat/hSLAM-M29F cells were generated by transfecting Jurkat cells with pCXN2-SLAM, pCXN2-SLAM-M29S, and pCXN2-SLAM-M29F, respectively, and were selected in RPMI supplemented with 10% FBS and 0.8 mg/ml geneticin. Three independent clones were generated for Jurkat cells expressing each hSLAM.

### Plasmid Construction

The expression vector, pCAG-SLAM, encoding the entire hSLAM open reading frame has been reported previously (Tatsuo et al., 2000b). The mutant SLAM (hSLAM-M29S and hSLAM-M29F)-expressing plasmids were generated by introducing specific mutations into the pCAG-SLAM plasmid by PCR-based site-directed mutagenesis using primers, 5'-AGGTGGGCGCTCTATGAACT-3' and 5'-AGTTCATAGAGCGCCCACCT-3' for hSLAM-M29S and 5'-GGTGGGCGCTTCATGAACTG-3' and 5'-GGTGGGCGCTTCATGAACTG-3' for hSLAM-M29F, respectively. The DNA fragments, hSLAM, hSLAM-M29S, and hSLAM-M29F, were inserted into the plasmid, pCXN2, which contains the neomycin resistance gene (Niwa et al., 1991). The generated plasmids were termed pCXN2-SLAM, pCXN2-SLAM-M29S, and pCXN2-SLAM-M29F. The canine SLAM-expressing plasmid, pCAG-dogSLAMtag, was described previously (Tatsuo et al., 2001). The pCAGGS vectors encoding the F and H proteins of the MV IC323 strain (pCA7-ICF and pCA7-ICH, respectively) were described previously (Tahara et al., 2007). Amino acid substitutions, F549S, F549N, F549H, F549Q, F549V, and F549I, were individually introduced into pCA7-ICH by PCR-based site-directed mutagenesis. pCAGGS vectors encoding the F and H proteins of the CDV Ac96I strain (pCA7-CDVF and pCA7-CDVH, respectively) were described previously (Otsuki et al., 2013). An amino acid substitution, P541S, was introduced into pCA7-CDVH by PCR-based site-directed mutagenesis. The full-length MV genome plasmid encoding enhanced green fluorescent protein (EGFP), p(+)MV323-EGFP, and support plasmids, pCITE-IC-N, pCITE-IC-PΔC, and pCITEko-9301B-L, were reported previously (Hashimoto et al., 2002; Seki et al., 2011). An amino acid substitution, F549S, was introduced into p(+)MV323-EGFP by PCR-based site-directed mutagenesis, generating p(+)MV323-EGFP-F549S.

### Viruses

MV IC323-EGFP strain was reported previously (Hashimoto et al., 2002). IC323-EGFP-F549S strain was generated from p(+)MV323-EGFP-F549S using a previously reported method (Seki et al., 2011). Working stocks of IC323-EGFP and IC323-EGFP-F549S strains were prepared using Vero/hSLAM cells and Vero.DogSLAMtag cells, respectively.

### Quantitative Cell Fusion Assay

A quantitative fusion assay was performed using a similar method to that described previously (Ishikawa et al., 2012). CHO/DSP_1–7_ cells were mixed with CHO/DSP_8–11_ cells at a ratio of 1:1 and the mixed cells were seeded in 24-well plates. The cells were transfected with mammalian cell expression plasmids encoding H protein (125 μg per well) and F protein (250 μg per well) of MV or CDV together with SLAM-expressing plasmid (125 μg per well) using FuGENE HD (using a 3:1 ratio of FuGENE HD transfection reagent to DNA). At 28 h post-transfection, *Renilla* luciferase activity was measured using the Renilla-Glo Luciferase assay system (Promega).

### Titration of Virus Stock

Monolayers of Vero.DogSLAMtag cells in six-well cluster plates were infected with serially diluted virus samples and incubated for 1 h at 37°C. The inoculum was then removed and the cells were washed with phosphate-buffered saline (PBS). The cells were overlaid with DMEM containing 5% FBS and 1% agarose. At 3 days post-infection (PI), the number of plaque forming units (PFU) was determined by counting the number of plaques under a fluorescence microscope.

### Virus Infection Assay

Vero.DogSLAMtag, Jurkat/hSLAM, Jurkat/hSLAM-M29S, and Jurkat/hSLAM-M29F cells were infected with serially-diluted virus samples (IC323-EGFP or IC323-EGFP-F549S) and incubated for 1 h at 37°C. The culture media were then supplemented with 10% FBS and 100 μg/ml of the fusion block peptide (Z-D-Phe-Phe-Gly; Peptide Institute Inc.; Richardson et al., 1980) to inhibit the second round of infection. At 1 day PI, the number of EGFP-expressing cells was counted under a fluorescence microscope. The number is expressed as cell infectious unit (CIU).

### Determination of the N-Terminal Sequence of hSLAM

The expression plasmid pCA7, encoding soluble hSLAM, which has its authentic signal peptide and a C-terminal 6x His-tag, was kindly provided by Dr. Y. Yanagi (Kyushu University; Hashiguchi et al., 2007). Subconfluent (~80%) monolayers of HEK293 cells were transfected with the plasmid using polyethyleneimine max (Polysciences). After transfection, the cells were maintained for 5 days in DMEM supplemented with 2% FBS. hSLAM secreted into the culture media was harvested and purified using Ni^2+^ affinity chromatography. Approximately 6 μg of protein was separated by SDS-PAGE using 10–20% gradient gels and transferred to polyvinylidene fluoride membranes using a semi-dry blotting system. Protein bands were visualized by staining with Coomassie Brilliant Blue, cut out and the amino acid sequence analyzed by standard Edman degradation.

### Homology Modeling

All the complex structures were constructed using the Molecular Operating Environment (MOE) program (Chemical Computing Group Inc.). The crystal structure of MV-H complexed with cottontop tamarin SLAM (PDB ID: 3ALW) was used as the initial template (Hashiguchi et al., 2011). To obtain additional information, another H protein structure (PDB ID: 2ZB6; Hashiguchi et al., 2007) was also used. A complex structure of MV-H (amino acids 152–606) IC-B strain (GenBank accession number NC_001498.1) and hSLAM (amino acids 21–137; GenBank accession number NM_003037) was modeled using the loop modeler utility of MOE. Missing hydrogen atoms were added using the Protonate 3D utility of MOE utilizing the Amber10:Extended Hückel Theory (EHT) force-field with solvation energy accounted *via* the Born model. The resultant complex structure was fully optimized with Amber10:EHT force-field. The structure was visualized using PyMOL (Molecular Graphics System, Version 2.0 Schrödinger, LLC.).

### Molecular Dynamics Simulation

The initial setups for the molecular dynamics (MD) simulations were made using the Amber 14 program (Case et al., 2014) utilizing the ff14SB force field (Maier et al., 2015). The constructed complex structure was solvated with a TIP3P (Jorgensen et al., 1983) water model in a 110 × 90 × 90 Å^3^ cubic box. Neutralizing counter ions were added to each system. Amber topology files created using Amber were converted to GROningen MAchine for Chemical Simulations (GROMACS) format using acpype.py script (Sousa da Silva and Vranken, 2012). All MD simulations were performed using the GROMACS package (Abraham et al., 2018). The bonds with H-atoms of the constructed structures were treated as rigid bodies by the LINCS algorithm (Hess et al., 1997). To equilibrate the system, an 800 ps isothermal-isobaric ensemble (NPT) simulation was performed using the Nose-Hoover thermostat (Nosé, 1984; Hoover, 1985) at 300 K by keeping heavy atoms constrained followed by a 100 ns NPT simulation with the Parrinello-Rahman method at 1 bar and 300 K (Parrinello and Rahman, 1981; Nosé and Klein, 1983). For non-local interactions, electrostatic interactions were calculated with the particle mesh Ewald method using a real space cutoff of 10 Å. The cutoff value for van der Waals interactions was set to 10 Å.

### Binding Free Energy Calculations

The molecular mechanics generalized Born surface area (MM-GB/SA) approach (Miller et al., 2012) implemented in Amber 14 was applied to calculate the binding free energy for all the simulated systems involved in our MD calculations. A total of 100 conformations were extracted from the last 20 ns of the MD simulations. The MM-GB/SA calculations were carried out after removing the water molecules and the counter ions. The entropy term (−TΔS) was computed under the Nmode module in Amber 14.

### Fragment Molecular Orbital (FMO) Calculation

Fragment molecular orbital (FMO) calculations were carried out using the PAICS program (Ishikawa et al., 2009). The correlated resolution-of-identity second-order Moller Plesset (RI-MP2) level of theory (Ishikawa et al., 2009) with correlation consistent double-zeta basis set cc-pVDZ was used for the calculations. Fragment assignment and PAICS input generation were performed using the PaicsView utility (Ishikawa, n.d.). In short, the FMO method is a first principles calculation-based quantum mechanical method. It is a powerful theoretical tool to reliably study protein-ligand and protein-protein interactions. In FMO calculations, each protein-ligand (or protein-protein) complex structure is divided into one-residue fragments, with cut-off points at Cα of each residue, with ligand (protein-ligand complex) also considered as a fragment and the properties of the whole system are derived in a many-body expansion by combining the properties of the fragments. By considering two-body systems (fragment pairs), the interfragment interaction energies (IFIEs) can be calculated, which is an important parameter for understanding the protein-ligand/protein-protein binding. In the present study, FMO was used to study the protein-protein interactions. FMO is an invaluable tool for the description of protein-ligand interactions (Minami et al., 2013; Tanaka et al., 2014; Otsuka et al., 2015; Arulmozhiraja et al., 2016; Fedorov and Kitaura, 2016; Heifetz et al., 2016; Yamamoto et al., 2018).

## Results

### *In silico* Analysis Shows MV-H Interaction With the ExNTR of hSLAM

**Figure 1:**
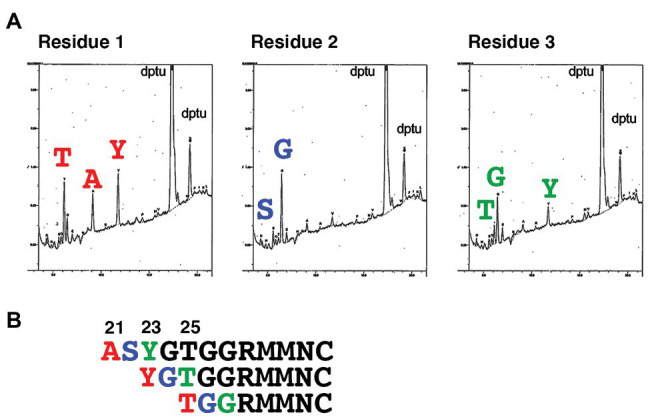
Determination of the N-terminal amino acid residues of human SLAM (hSLAM). **(A)** The left, middle, and right panels show amino acid profiles of the first, second, and third cycles of Edman degradation, respectively. **(B)** The extreme N-terminal amino acid sequence of hSLAM. The three patterns of the N-terminal sequence are shown. The first, second, and third residues are shown in red, blue, and green, respectively.

**Figure 2:**
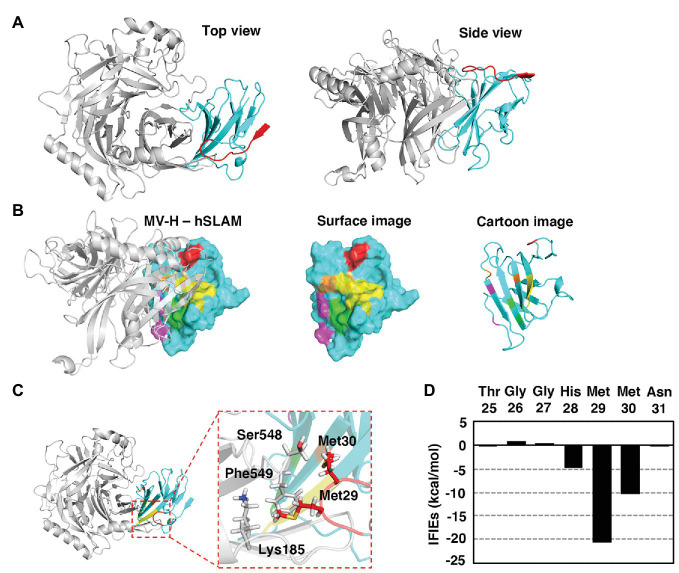
Modeled MV H protein (MV-H) and hSLAM complex structure. **(A–C)** The MV-H (amino acids 152–606) and hSLAM (amino acids 21–137) complex structure was modeled using the Molecular Operating Environment (MOE) program. A reported structure (PDB ID: 3ALW; Hashiguchi et al., 2011) was used as the initial template. Missing hydrogen atoms were added using the Protonate 3D utility of MOE utilizing the Amber10:extended Hückel theory (EHT) force-field with solvation energy accounted for *via* the Born model. The resultant complex structure was optimized with Amber10:EHT force-field. The structure was visualized using PyMOL. **(A)** The overall complex structure of MV-H (gray) and hSLAM (cyan). The extreme N-terminal region (ExNTR) of hSLAM is shown in red. **(B)** The previously identified four binding components (sites 1–4; Hashiguchi et al., 2011) are shown in magenta, light green, yellow, and orange. The newly identified interaction using ExNTR of hSLAM is shown in red. **(C)** The modeled structure showed that hSLAM-Met29 and hSLAM-Met30 in the loop domain of hSLAM ExNTR interact with MVH-Lys185, MVH-Ser548, and MVH-Phe549. In particular, the interaction of hSLAM-Met29 was established with MVH-Phe549 through a CH-π interaction. **(D)** Fragment molecular orbital (FMO) interaction energies calculated between hSLAM-ExNTR residues and MV-H. Interfragment interaction energies (IFIEs) of a SLAM residue denote the sum of its interactions with all the MV-H residues.

SLAM is a type I transmembrane protein that possesses a signal peptide at the N terminus. N-terminal analysis of hSLAM by standard Edman degradation detected three different N-terminal patterns (Figure 1). The N-terminal residues of the three hSLAM patterns were alanine, tyrosine, and threonine at positions 21, 23, and 25, respectively (Figure 1). A crystal structure of MV-H (Edmonston vaccine strain) complexed with cottontop tamarin SLAM (Hashiguchi et al., 2011) has been reported previously, but several residues or certain domains, including the ExNTR of SLAM, were not visualized in the structure. Therefore, a complete complex structure of MV-H (wild-type IC-B strain; amino acids 152–606) and hSLAM (amino acids 21–137) was modeled in this study (Figure 2A, Supplementary Material 1). MD simulations showed that the complex was structurally stable and the binding free energy in the complex was −21.2 kcal/mol. The data showed that, in addition to the four binding components (sites 1–4) between SLAM and MV-H, which were identified previously (Hashiguchi et al., 2011), the ExNTR of hSLAM formed an effective interaction with MV-H (Figures 2B,C). The interaction energy obtained between the loop domain of hSLAM ExNTR and MV-H using the first principles calculation-based FMO computations revealed that among the ExNTR residues, a methionine residue at position 29 (hSLAM-Met29) interacted with MV-H most strongly (Figures 2C,D). The modeled structure showed that hSLAM-Met29 and hSLAM-Met30 (a methionine at position 30) in the loop domain of hSLAM ExNTR interact with lysine, serine, and phenylalanine at positions 185, 548, and 549 (MVH-Lys185, MVH-Ser548, and MVH-Phe549). The interaction of hSLAM-Met29 was established with MVH-Phe549 through a CH-π interaction (Figure 2C).

### The hSLAM-Met29 and MVH-Phe549 Residues Are Important for MV-H and hSLAM-Mediated Cell-Cell Fusion

**Figure 3:**
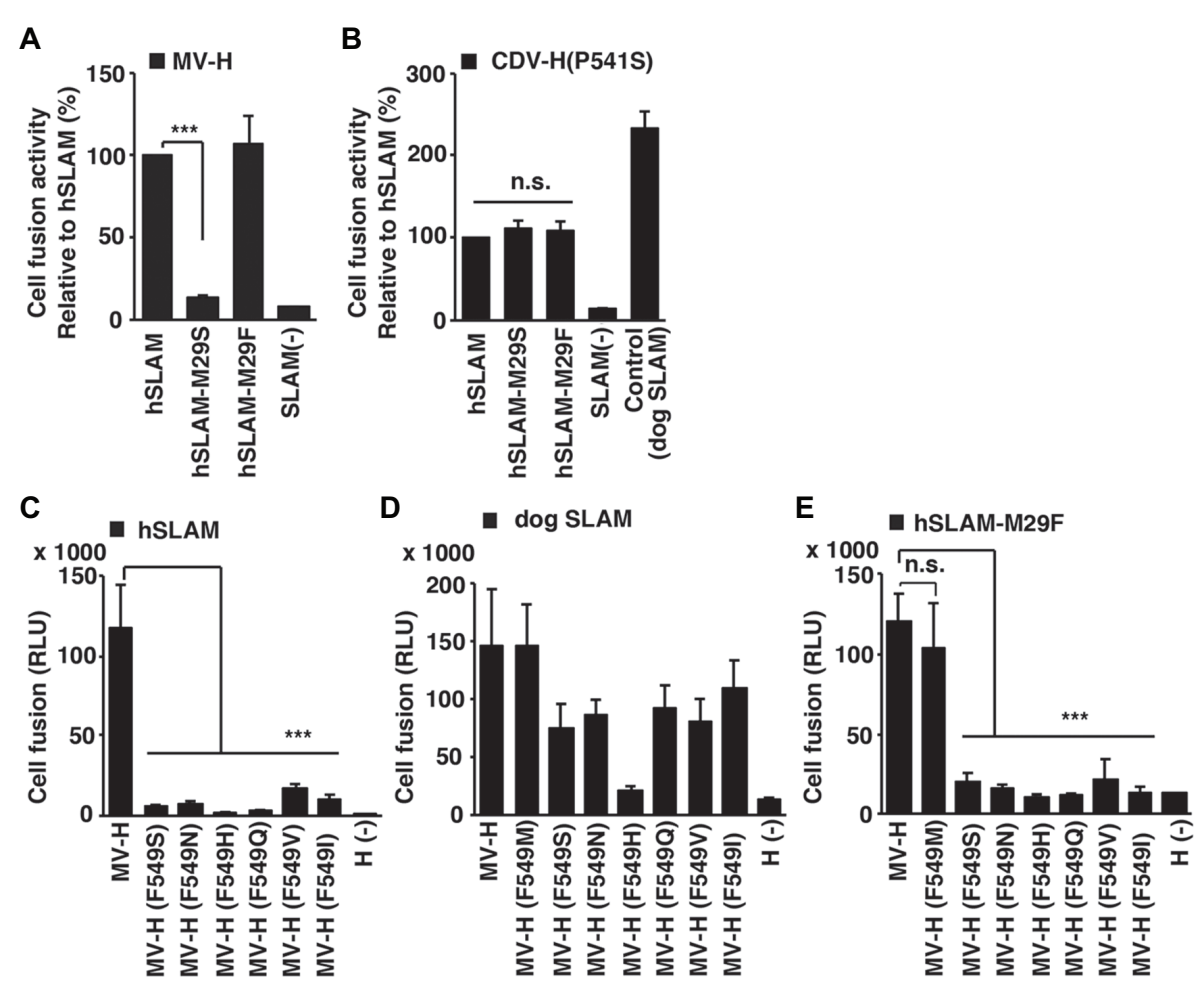
Importance of hSLAM-Met29 and MVH-Phe549 shown by cell-cell fusion assays. **(A–E)** A quantitative cell fusion assay using the dual split protein (DSP) system. **(A)** Cells were transfected with MV-H and MV-F expression plasmids together with a non-mutated hSLAM‐ or mutated (M29S or M29F) hSLAM-expressing plasmid. **(B)** Cells were transfected with canine distemper virus (CDV)-H/P541S and CDV-F expression plasmids together with a non-mutated hSLAM-, mutated (M29S) hSLAM-, or dog SLAM-expressing plasmid. **(C–E)** Cells were transfected with non-mutated or mutated (F549S, F549N, F549H, F549Q, F549V, F549I, or F549M) MV-H and MV-F expression plasmids together with **(C)** an hSLAM-expressing plasmid, **(D)** a dog SLAM-expressing plasmid, or **(E)** a mutated (M29S) hSLAM-expressing plasmid. **(A,B)** For control cells [SLAM (−)], the SLAM-expression plasmid was omitted from the transfection mixture. **(C–E)** For control cells [H (−)], the MV-H-expression plasmid was omitted from the transfection mixture. At 1 day post transfection, the *Renilla* luciferase activity was measured. Error bars indicate the standard deviations of triplicate wells. **(A–E)** ^***^ *p* < 0.001; ns, not significant.

The fragment molecular orbital interaction energies obtained between the loop domain of hSLAM ExNTR and MV-H revealed that hSLAM-Met29 was the most influential residue among the hSLAM ExNTR residues (Figure 2D). hSLAM-Met29 interacts with MVH-Phe549 through a CH-π interaction (Figure 2C). Cell-cell fusion assays were performed to assess the MV-H and hSLAM interaction. When hSLAM-Met29 was substituted with a serine (M29S), the fusion activity was reduced greatly (Figure 3A), indicating the importance of the CH-π interaction between hSLAM-Met29 and MVH-Phe549. To show the integrity of the mutated hSLAM with M29S (hSLAM/M29S), a mutant CDV strain (CDV/P541S) having a hSLAM-using ability (Sakai et al., 2013) was used. Non-mutated hSLAM and mutated hSLAM/M29S were equally functional as a CDV/P541S receptor (Figure 3B). These data indicated that the hSLAM-Met29 residue was specifically important for the interaction with MV-H. When hSLAM-Met29 was substituted with phenylalanine (M29F), hSLAM retained full functionality as an MV receptor (Figure 3A). It was most likely that a newly established π-π interaction between hSLAM-Phe29 and MVH-Phe549 supported the hSLAM-MV-H interaction as the original CH-π interaction. The MVH-Phe549 residue was predicted to interact with hSLAM-Met29 (Figure 2C). To show the importance of MVH-Phe549, the residue was substituted with several different amino acid residues (serine, F549S; asparagine, F549N; histidine, F549H; glutamine, F549Q; valine, F549V; and isoleucine, F549I). All the mutant MV-Hs showed greatly reduced fusion activities (Figure 3C), indicating the important role of MVH-Phe549 in the interaction with hSLAM. In contrast, MVH-Phe549 was not critical for the interaction with dog SLAM, because MV-H with F549S, F549N, F549Q, F549V, or F549I showed moderate levels (52–76%) of cell-cell fusion, when compared with the non-mutated MV-H (Figure 3D). Thus, these data indicated that the CH-π interaction between SLAM-Met29 and H-Phe549 residues was only important for hSLAM. Methionine is not in that position in cow, sheep, dolphin, seal, or dog SLAMs; leucine or serine was present instead (Takeda et al., 2020). To show the importance of the CH-π interaction between these residues further, the amino acids of these positions of hSLAM and MV-H were exchanged with each other; that is, M29F and F549M substitutions were introduced into hSLAM and MV-H, respectively. The data clearly showed that MV-H with F549M (MV-H/F549M) successfully used hSLAM with M29F (hSLAM/M29F; Figure 3E). By contrast, the use of hSLAM/M29F by other mutant MV-H proteins with F549S, F549N, F549H, F549Q, F549V, or F549I was inefficient (Figure 3E).

### hSLAM-Met29 and MVH-Phe549 Residues Enhance hSLAM-Dependent MV Infection

**Figure 4:**
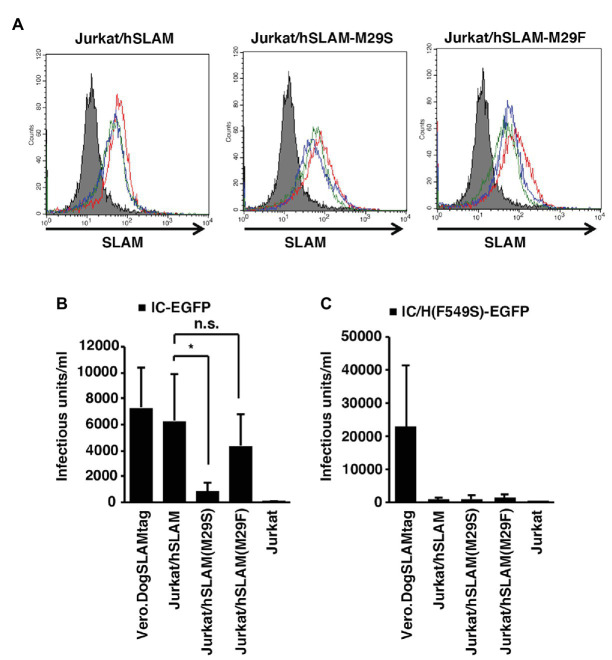
Importance of Met29^hSLAM^ and Phe549^MVH^ shown by virus infection assays. **(A)** SLAM expression profile of the parental Jurkat, Jurkat/hSLAM, Jurkat/hSLAM-M29F, and Jurkat/hSLAM-M29S cells. Three independent clones of Jurkat cells expressing each hSLAM were generated. The parental Jurkat cells (filled gray profile) and three individual clones (red, blue, and green empty profiles) were stained with the anti-SLAM monoclonal antibody (IPO-3) followed by an Alexa Fluor 546-conjugated anti-mouse IgG. **(B,C)** The parental Jurkat, Jurkat/hSLAM, Jurkat/hSLAM-M29F, Jurkat/hSLAM-M29S, and Vero.DogSLAMtag cells were infected with IC323-enhanced green fluorescent protein (EGFP) **(B)** or IC323-EGFP-F549S **(C)** and cultured with the fusion block peptide. At 1 day post-infection (PI), the cell infectious unit (CIU) was determined by counting the number of EGFP-expressing cells. **(B,C)** ^*^ *p* < 0.03; ns, not significant.

To show the important roles of hSLAM-Met29 and MVH-Phe549 in the context of virus infection, cell lines constitutively expressing mutated hSLAM (M29S and M29F) or non-mutated hSLAM were generated. For these experiments, we used Jurkat cells, a human T cell-derived cell line that does not express SLAM (Tatsuo et al., 2000a). The cell lines were named Jurkat/hSLAM-M29S, Jurkat/hSLAM-M29F, and Jurkat/hSLAM, respectively. The expression levels of SLAM in these cell lines were similar (Figure 4A). Infection of wild-type MV IC323-EGFP (Takeda et al., 2000; Hashimoto et al., 2002) was enhanced by expressing any of the SLAMs but notably this enhancement was ~10-fold greater in Jurkat/hSLAM and Jurkat/hSLAM-M29F cells compared with that in Jurkat/hSLAM-M29S cells (Figure 4B). These data showed that Met29 in hSLAM or Phe29 in hSLAM/M29F enhanced the MV infection by ~10-fold likely by interacting with MVH-Phe549. The infectivity in Jurkat/hSLAM was similar to that in Vero.DogSLAMtag cells (Figure 4B). A recombinant MV possessing an F549S mutation (IC323-EGFP/F549S) was generated. It was expected that no interaction could be established between MVH-Ser549 and any of the position 29 hSLAM resides. As expected, infectivity of IC323-EGFP/F549S was significantly lower in all the hSLAM expressing cells (Jurkat/hSLAM, Jurkat/hSLAM-M29S, or Jurkat/hSLAM-M29F) compared with that in Vero.DogSLAMtag cells (Figure 4C). These data demonstrated that both hSLAM-Met29 and MVH-Phe549 were important for MV infection using hSLAM.

## Discussion

Morbilliviruses (MV, RPV, PPRV, CeMV, PDV, and CDV) commonly use SLAM and PVRL4 as receptors, but each morbillivirus has a clear host range. Amino acid sequence variations among host animal SLAMs in part explain the host range difference among morbilliviruses. However, in many cases, non-host animal SLAMs, such as dolphin, seal, and dog SLAMs, function as efficient receptors for different morbilliviruses (Takeda et al., 2020). Exceptionally, hSLAM only functions efficiently for MV (Takeda et al., 2020). In this study, the structural and molecular basis for MV-H interaction with hSLAM was studied in further detail.

First, the N-terminus of hSLAM was determined. SingalP software^1^ predicted that the first 20 amino acids of hSLAM function as a signal peptide. However, Edman degradation analysis showed three different patterns of the N-terminal sequence. Ambiguous or multiple cleavage sites of a signal peptide are rare but one example is reported for bovine pituitary growth hormone (Lingappa et al., 1977). The three positions met the (−3, −1) rule for the signal peptide cleavage site (Von Heijne, 1983, 1984) but prediction of sequence requirement of eukaryotic signal peptide cleavage sites is very difficult because they accept a number of different amino acids and have no obvious pattern (Nielsen et al., 1997). Although this ambiguity of the SLAM N-terminus may need further assessment, the same three N-terminal patterns were obtained using macaque SLAM (GenBank accession number XM_001117605; data not shown), having 98.33% homology to hSLAM.

The complex structure of MV-H and SLAM (PDB ID: 3ALW) determined by Hashiguchi et al. (2011) provides great insight into our understanding of SLAM recognition by morbilliviruses, but several residues or certain domains were not visualized in the structure, in part because of the flexibility of these domains and artificial modification of expressed proteins for crystallization. Also, cottontop tamarin SLAM and not hSLAM was used to determine the structure. Therefore, our next step was to obtain the complete complex structure of hSLAM and MV-H. The longest version of the N-terminal sequence (the first amino acid is alanine at position 21) was used for the structure modeling. The previously determined MV-H and SLAM complex structure (PDB ID: 3ALW; Hashiguchi et al., 2011) is composed of the MV-H head (amino acids 184–607) and the cottontop tamarin SLAM V (amino acids 30–140) domains, which are linked by a 12-residue flexible linker (Gly-Gly-Gly-Ser)_3_. Thus, the protein lacks up to 10 amino acid residues (amino acids 21–29) from the ExNTR of hSLAM. Additional structural information was obtained using another H protein structure (PDB ID: 2ZB6; Hashiguchi et al., 2007), which is composed of the head, a part of the stalk region, and the connecting loop region between the head domain and stalk region (amino acids 149–617). However, a large part (amino acid positions 167–183) of the loop region connecting the head domain and the stalk region was not visualized in the structure, likely because of the flexibility of the region (Hashiguchi et al., 2007). The missing regions were modeled in this study and the completed complex structure demonstrated a previously unidentified interaction between MV-H and the ExNTR of hSLAM.

Importantly, our data using expressed proteins and infectious viruses demonstrated that this interaction (between hSLAM-Met29 and MVH-Phe549) is indeed important for MV infection using hSLAM. A following important question was whether this interaction using SLAM ExNTR is specific for MV-hSLAM or generally observed for morbillivirus infection using their host animal SLAMs. None of cow, sheep, dolphin, seal, or dog SLAMs have methionine at this position (Met29); they have leucine or serine. Thus, Met29 is unique for hSLAM. However, our data cannot exclude the possibility that other morbilliviruses have a similar or different type of interaction with the ExNTR of their host animal SLAM. To avoid over-interpretation, we emphasize that the V domain β-sheet of SLAM establishes the primarily interaction with morbillivirus H (Hashiguchi et al., 2011), and that interaction with SLAM ExNTR may not be required for morbillivirus infection using SLAM. We rather suggest that MV has uniquely established this additional interaction to use hSLAM. From an evolutionary point of view, MV has a unique host among morbillivirus host animals (Takeda et al., 2020). All other host animals of morbilliviruses are classified in *Cetartiodactyla* and *Carnivora* orders but not in the *Primate* order (Takeda et al., 2020). MV has likely evolved from an ancestral bovine virus (RPV-like virus) *via* a dramatic host switching event across orders (Takeda et al., 2020). If the hSLAM-Met29 and MVH-Phe549 interaction has played a critical role in this host switching event, RPV-H may acquire hSLAM-using ability by having Phe549 in its H protein. RPV-H has serine but not phenylalanine at this position. This possibility was tested using our DSP system. The serine to phenylalanine change (S549F) in RPV-H did increase hSLAM-using ability but only slightly (data not shown). It is likely that mutations in the major binding site, the V domain β-sheet of hSLAM, were also required during the evolution from an ancestral bovine virus to a human virus to establish effective interaction with hSLAM.

In conclusion, the complex structure of MV-H and hSLAM was studied in detail by *in silico* analyses. These analyses identified a unique interaction between MV-H and hSLAM in addition to the known binding components, sites 1–4. The interaction was formed using the ExNTR of hSLAM and the key residue in hSLAM was Met29, which was predicted to establish a CH-π interaction with MVH-Phe549. Our data using genetically engineered cell lines and recombinant viruses showed that the hSLAM-Met29 and MVH-Phe549 interaction enhances hSLAM-dependent MV infection. We speculate that this interaction may have contributed to MV adaptation to humans because this interaction enables efficient use of hSLAM, which is unique for MV.

## Data Availability Statement

The datasets presented in this study can be found in online repositories. The names of the repository/repositories and accession number(s) can be found in the article/Supplementary Material.

## Author Contributions

FS, KO, TM, KM, HT, and MT designed the study. FS, YY, and HF performed experiments. FS, YY, HF, KO, TM, KM, HT, and MT analyzed the data. FS, YY, HF, KM, HT, and MT wrote the manuscript. All authors reviewed the manuscript. All authors contributed to the article and approved the submitted version.

### Conflict of Interest

The authors declare that the research was conducted in the absence of any commercial or financial relationships that could be construed as a potential conflict of interest.

## Footnotes

^1^http://www.cbs.dtu.dk/services/SignalP/

## Supplementary Material

The Supplementary Material for this article can be found online at:

https://www.frontiersin.org/articles/10.3389/fmicb.2020.01830/full#supplementary-material.

## Abbreviations

MV: Measles virus
SLAM: Signaling lymphocytic activation molecule
PVRL4: Poliovirus receptor-like 4
H: Hemagglutinin
F: Fusion
RPV: Rinderpest virus
PPRV: Peste des petits ruminants virus
CeMV: Cetacean morbillivirus
PDV: Phocine distemper virus
CDV: Canine distemper virus
hSLAM: Human SLAM
MV-H: MV H protein
ExNTR: Extreme N-terminal region
DMEM: Dulbecco’s modified Eagle’s medium
FBS: Fetal bovine serum
DSP: Dual split protein
EGFP: Enhanced green fluorescent protein
PBS: Phosphate-buffered saline
PI: Post-infection
PFU: Plaque forming unit
CIU: Cell infectious unit
MOE: Molecular Operating Environment
MD: Molecular dynamics
MM-GB/SA: Molecular mechanics generalized Born surface area
FMO: Fragment molecular orbital
RI-MP2: Resolution-of-identity second-order Moller Plesset
IFIEs: Interfragment interaction energies
